# COVID-19 stigma

**DOI:** 10.1192/j.eurpsy.2021.716

**Published:** 2021-08-13

**Authors:** G. Marinho, J. Peta, J. Pereira, M. Marguilho

**Affiliations:** 1 Clinica 6, CHPL, Lisbon, Portugal; 2 Psychiatry, Centro Hospitalar Psiquiátrico de Lisboa, Lisboa, Portugal; 3 Clínica 5, Centro Hospitalar Psiquiátrico de Lisboa, Lisboa, Portugal

**Keywords:** infectious diseases, Stigma, COVID-19

## Abstract

**Introduction:**

‘Health‐related stigma’ is typically known as social rejection or exclusion of individuals and populations suffering from specific health problems. Results on previous infectious diseases showed that stigma can be experienced by survivors but also by health‐care workers (HCW). Several factors contribute to stigma associated with infectious diseases, such as people’s knowledge, myths and stories transmitted by the mass and social media and psychosocial variables, such as risk perception and fear of being infected. COVID‐19 is a new disease with many unknown aspects and, naturally, people are afraid of the unknown.

**Objectives:**

To reflect on infectious diseases and social stigma during covid-19 pandemics.

**Methods:**

Pubmed and Google Scholar search.

**Results:**

Stigmatization can considerably increase psychosomatic distress and disturbance and can negatively affect people with infection and those at risk of infection in seeking medical care. HCWs and volunteers working in the field may also become stigmatized, leading to higher rates of distress, stress, and burnout When people avoid groups or geographic areas related to infectious diseases, this can pose significant economic losses. Thus, stigma is more than a mere negative outcome of infectious diseases; it is both a factor that contributes to the epidemics and pandemics and a disease in itself.

**Conclusions:**

Anticipating disease‐related stigma during the COVID‐19 pandemic enables policy‐makers to address it, restricting its adverse effects. The hidden burden caused by this stigma can cause severe consequences for patients, HCW, and public health measures, so, coordinated psychological interventions to overcome this crisis seems essential.

